# PEDOT-Based Conducting Polymer Actuators

**DOI:** 10.3389/frobt.2019.00114

**Published:** 2019-11-19

**Authors:** Faqi Hu, Yu Xue, Jingkun Xu, Baoyang Lu

**Affiliations:** School of Pharmacy, Jiangxi Science and Technology Normal University, Nanchang, China

**Keywords:** conducting polymer, PEDOT, PEDOT:PSS, actuator, artificial muscle

## Abstract

Conducting polymers, particularly poly(3,4-ethylenedioxythiophene) (PEDOT) and its complex with poly(styrene sulfonate) (PEDOT:PSS), provide a promising materials platform to develop soft actuators or artificial muscles. To date, PEDOT-based actuators are available in the field of bionics, biomedicine, smart textiles, microactuators, and other functional applications. Compared to other conducting polymers, PEDOT provides higher conductivity and chemical stability, lower density and operating voltages, and the dispersion of PEDOT with PSS further enriches performances in solubility, hydrophility, processability, and flexibility, making them advantageous in actuator-based applications. However, the actuators fabricated by PEDOT-based materials are still in their infancy, with many unknowns and challenges that require more comprehensive understanding for their current and future development. This review is aimed at providing a comprehensive understanding of the actuation mechanisms, performance evaluation criteria, processing technologies and configurations, and the most recent progress of materials development and applications. Lastly, we also elaborate on future opportunities for improving and exploiting PEDOT-based actuators.

## Introduction

Muscles are the indispensable constituents of the human body, ranging from information transfer to energy supply and transmission; thus, there is tremendous interest in their related artificial products like “artificial muscles.” Nowadays so-called artificial muscles are utilized as electroactive polymer (EAP) actuators in medicine, bionics, cell biology, and microelectromechanical systems (Jager et al., 2000; Otero et al., 2012; Agrawal et al., 2019; Jayathilaka et al., 2019). The actuator is considered as a device that can achieve the conversion from external stimuli (such as electrical, thermal, optical energy) to mechanical formalization (Panda and Acharya, 2019); such devices could therefore be developed into “artificial muscles,” but it is a challenging task. In the 1950s, McKibben et al. first reported a pneumatic actuator as a powertrain for artificial muscles, but such actuators were bulky and limited by the auxiliary system (Klute et al., 1999). Generators and hydraulic systems are also limited by bulky volumes, and their flexibility, strength, and overall working ability are far from those of human muscles. Subsequently, shape memory alloys (SMAs) (Huang, 2002) and electroactive ceramics (EACs) (O'Halloran et al., 2008) were used as artificial muscle materials, but they still suffered from the shortcomings of slow response speed, unpredictable movement, limited size, large brittleness, small strain capabilities, and high power consumption.

Until later, various electroactive polymers (EAPs) were used to fabricate actuating devices that could generate physical deformation under the action of the current, voltage, or electric field, and they are distinguished by their ability to convert electrical energy into mechanical energy (Bar-Cohen and Anderson, 2019). In comparison to electroactive ceramics and shape memory alloys, see Halloran's work for details (O'Halloran et al., 2008), actuators fabricated by EAPs can present lower driving potential and are easier to miniaturize. EAPs are the current promising artificial biological muscles, owing to their high resilience and fracture toughness, ability to engender large actuation strains, and inherent vibration-damping properties, which are referred to as artificial muscles in many reports (Bar-Cohen, 2000, 2002; Asplund et al., 2010). According to the deformation mechanism, EAPs can be divided into two categories (Smela, 2003; Romasanta et al., 2015; Kongahage and Foroughi, 2019; Melling et al., 2019). One is the electric field active material (also known as electronic EAPs), which is driven by electric fields or electrostatic action (Coulomb force) in dry environments. Their actuation typically requires high voltage (about 20 V μm^−1^) (Melling et al., 2019) and specialized electronic equipment, which limits certain applications. The other current-active material is ionic EAPs driven by the mobility or diffusion of ions in solution or air environment. These are typically composed of ionic polymer gels, carbon nanotubes, ionic polymer-metal composites, and conducting polymers, which can be utilized to create soft and small actuators operating under low voltages (typically 1–7 V) (Kongahage and Foroughi, 2019). Among the ionic EAP actuators, ionic polymer metal composite (IPMC) and conductive polymer (CP) actuators have been widely studied (Kruusamäe et al., 2015). IPMCs can be driven at a voltage range (typically 1–4 V) and produce strains (about 0.5–3.3%). By contrast, CPs can be driven at a smaller voltage (typically 1-3 V) and provide a larger strain range (0.5%–12%) at the same time (Madden et al., 2004). Moreover, the more unique properties of CPs, such as holding strain under DC voltage or at open circuit, make it easy to produce consistent material and miniaturization. All these advantages make them promising materials for soft actuators (Melling et al., 2019).

Among the conducting polymers, only a few are used to design actuators. Material selection is mainly focused on polypyrrole (PPy), which has been fully studied and can be used in large-scale production (Berdichevsky and Lo, 2006; Kiefer et al., 2007). The PPy has advantages that can generate large actuation strain and stress and easily deposit dense or porous gel films in aqueous solutions or organic electrolytes (Hara and Takashima, 2004; Zainudeen et al., 2008; Temme et al., 2012). However, PPy also suffers from several drawbacks, such as high rigidity, low conductivity, ion diffusion rate, and the risk of over-oxidation (Madden et al., 2001; Temmer, 2013). Poly(3,4-ethylenedioxythiophene) (PEDOT) has been extensively studied for its high, stable, and tunable electrical conductivity (Wang, 2009). Its conductivity in the doped state is one order of magnitude higher than that of PPy and can be further improved by blending or post-treatment (Gueye, 2016). Besides, the highly stable oxidized state enables it to maintain conductivity for several months even in high temperature, and thus would considerably improve the cycling stability of actuators, which makes it an excellent choice for actuator electrode materials (Temmer, 2013). Meanwhile, the synthesis of PEDOT is simpler and more controllable either by chemical or electrochemical methods, while PPy synthesized by chemical methods is usually powder, insoluble in water or organic solvents (Joo et al., 2000; Ha et al., 2004). Also, the ease of structural modification on thiophene ring, ethylene, and dioxy group and also the main backbone can readily obtain numerous PEDOT analogues (Chen et al., 2012; Lu et al., 2013; Feng et al., 2015), derivatives (Ming et al., 2015b; Lin et al., 2017), and it-based copolymers (Lu et al., 2014; Ming et al., 2015a). In addition, dispersing PEDOT with PSS to form PEDOT:PSS aqueous solution improves the solubility, hydrophilicity, processability, and flexibility of PEDOT materials, further enriching their application in actuator areas (Lu et al., 2019; Rohtlaid, 2019; Yuk et al., 2019).

Starting with the work of Inganäs and coworkers, experiments with electrical stimulation of PEDOT film have shed light on the deformation mechanism of the freestanding polymer film, leading to the development of PEDOT actuators (Chen et al., 1996). To date, many researchers have focused their attention on the actuators fabricated by PEDOT and PEDOT:PSS and have made great effort to promote their development. PEDOT-based actuators have developed into many configurations, such as self-supporting thin films, double or three-layer composite thin films, and fiber or film micro-actuators, which can realize linear, bending, or buckling in solution or air. Plenty of studies have been devoted to blending or doping PEDOT or PEDOT:PSS electrodes with other polymers, inorganic carbon materials, or developing novel polymer electrolytes to improve the electrochemical and mechanical properties of the actuators. PEDOT-based actuators with high stress (up to 50 MPa) (Wang et al., 2017) and strain (up to 2%) (Vandesteeg et al., 2003), low voltage (typically 1–3 V) (Kotal et al., 2016), and a long cycle life (up to 10^6^ cycles) (Vidal et al., 2004) have therefore been developed. While commendable advances are presented in actuators based on PEDOT and PEDOT:PSS, some inherent limitations remain as big challenges, e.g., low strain, poor cycle life, and a need for an electrolyte source, etc. Thus, a deeper understanding of the structure-performance relationship of these materials is vital for the future development of PEDOT-based actuators. In this review, we provide a brief overview of the advancements of PEDOT-based conducting polymer actuators and discuss fundamental actuation mechanisms, configurations, evaluation metrics of actuation performances, and material properties in detail. On this basis, advances in materials, manufacture technology, and application are reviewed. We also point out the remaining challenges and opportunities and hope to bring new insights for the further development of PEDOT-based actuators.

## Actuation Mechanisms

The key to the actuation behavior of CP's actuator depends on the volume change of polymer film electrodes during the electrochemical doping/dedoping process. When applying a voltage, electrons will be lost or accepted in the conductive polymer chain. In order to maintain the charge neutrality in the whole structure, anions and cations in the electrolyte will migrate between the polymer matrix and the surrounding electrolyte environment, resulting in the expansion or contraction of the polymer film electrodes, causing the geometric deformation of the whole actuator (Melling et al., 2019). On the other hand, CPs like PEDPT: PSS have a porous structure and easily absorb or desorb water; thus, changing the water content can also cause the expansion or shrinkage of the polymer film. The ions doping/dedoping mechanism of p-doped polymer based on PEDOT and the moisture sorption/desorption mechanism based on PEDOT:PSS are discussed as below.

### Ions Doping/Dedoping Mechanism

PEDOT is typically produced by an oxidative polymerization process. During the oxidation process, with the removal of electrons, the crosslink behavior formed between the generated polarons and dopant anions and the insertion of anions causes the PEDOT film to be swollen in the oxidized state. When a negative voltage is applied, depending on the size of the neutralized anions in the polymer chain, there are two ways of anion migration or cation migration to occur in the reduction process, which leads to shrinkage or expansion of the PEDOT film in the reduced state (Bay et al., 2001; Bund and Neudeck, 2004; Yang and Zoski, 2006).

**Figure 1:**
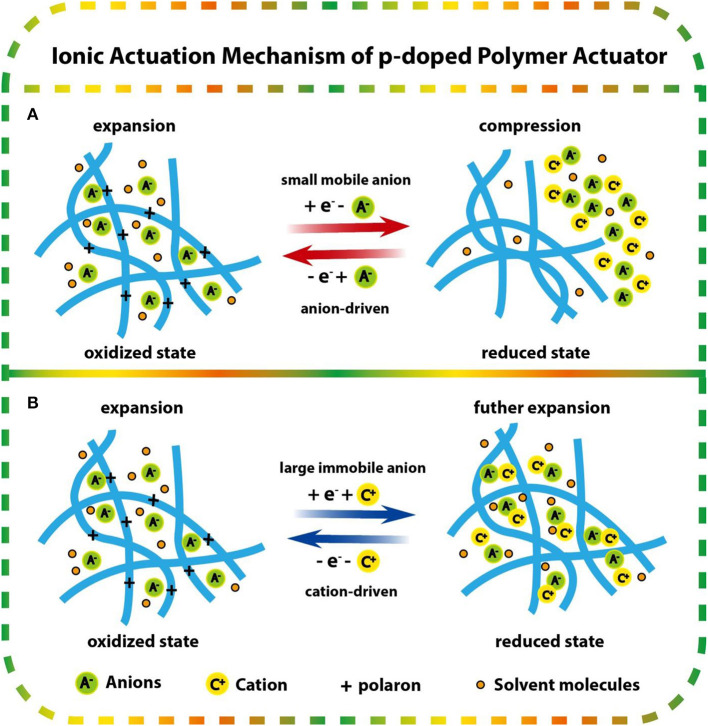
Ionic actuation mechanism of p-doped polymer actuators: **(A)** anion transport dominated when the anion was small and highly mobile, **(B)** cation transport dominated when the anion was large and immobile.

When the dopant anion size in the PEDOT chain is small and mobile (Figure 1A), such as *p*-toluenesulfonic acid (PTSA), through applying a negative voltage, anions and solvent molecules can be easily expelled to maintain the neutralization of the PEDOT film, which causes the film to shrink in the reduced state. Oxidation occurred when voltage reversal, anions, and solvent molecules in the electrolyte move in, and the PEDOT film expands again in the oxidation state. The redox process is dominated by anion migration, and so it is called “anion-driven.” When the dopant anions in the PEDOT chain are large and immobile (Figure 1B), such as polystyrene sulfonate (PSS), they become wrapped in the polymer matrix during the synthesis process and cannot leave during the reduction. At this time, cations migrate from the solution to the PEDOT chain to neutralize anions and negative charges, which causes the film to expand in the reduced state. Applying positive voltage again, cations are expelled and the PEDOT film shrinks in the oxidation state. The redox process is dominated by cation migration, so it is called “cation-driven.”

Obviously, the type of electrolyte used in the PEDOT oxidation polymerization and actuation process has a significant effect on ion migration. It has been reported that when using molecular electrolyte solution, unless the doped anions are very large, such as with the polystyrene sulfonic acid anion (PSS^−^) or dodecylbenzenesulfonic acid anion (DBS^−^), the ion migration process of PEDOT actuators is mainly driven by anions. On the other hand, when using ionic liquid electrolytes diluted in organic solutions, such as 1-ethyl-3-methyl-imidazolium bis(trifluoromethyl-sulfonyl)imide (EMIBTI), the ion migration process also presents typical anion-driven characteristics. While in the neat EMIBTI, the ion migration process presents cation-driven characteristics dominated by imidazole ions (Irvin and Carberry, 2013). Moreover, the dimensional changes of the actuators not only rely on the ion flux transmission between polymer chains; the exchange of solvent molecules accompanying ion migration and the conformational change of the polymer backbone also play a role, as detailed in the works of Bund and Neudeck (2004) and Chen et al. (1996).

### Moisture Sorption/Desorption Mechanism

Based on the conductivity and hydrophilicity of PEDOT:PSS and the photothermal properties of PEDOT, using them as an active layer to laminate with an inert layer fabricated actuators that can respond to stimulus such as electrical, thermal, or near infrared (NIR) light. When current or NIR light passes through the polymer film, the temperature of the film rises due to *Joule* heating, which leads to the desorption of water molecules in the film and, eventually, shrinkage. When the current is removed, the film absorbs water vaper from ambient air and expands again. When NIR light is turned off, the film is cooled and restored to its original state. The stress gradient between the active electrode layer and the inert layer drives the actuator to bend.

**Figure 2:**
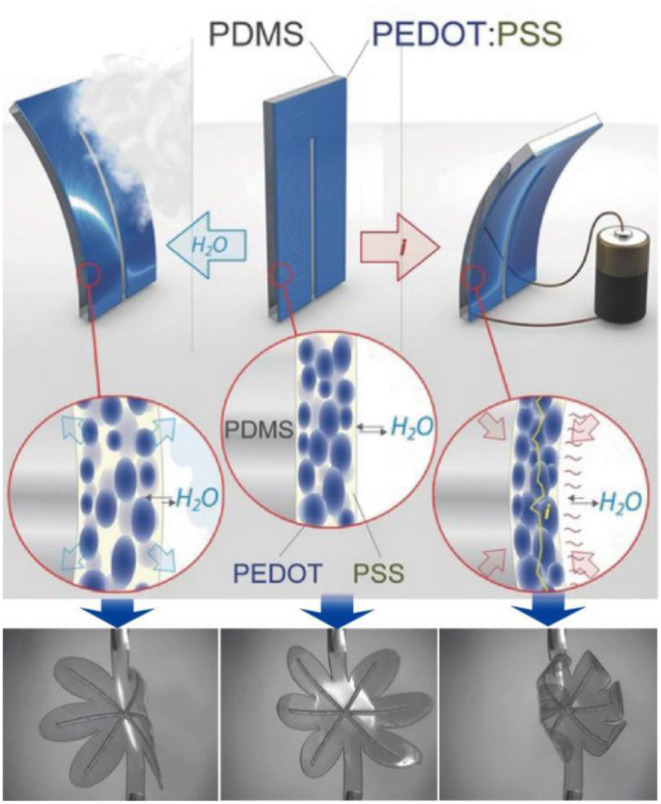
Schematic representation of the working principle behind the PEDOT/PDMS actuator based on the sorption/desorption of environmental moisture, using the actuation movement of an electrically driven flower-shaped sample as an example. Reproduced with permission (Temmer, 2013). Copyright 2018, Wiley.

For example, Okuzaki et al. (Asaka and Okuzaki, 2012) first investigated the humidity sensitive PEDOT:PSS film as a soft actuator (Okuzaki et al., 2009b) whose actuation was achieved through the absorption of moisture from high pressure water vapor and which underwent constriction due to the *Joule* heat generated by the current. After that, Taccola and coworkers (Taccola et al., 2015) reported a bilayer, humidity-response actuator that was composed of an active PEDOT:PSS layer and an inert PDMS substrate, and its anisotropic motion was dependent on the stress gradient between the active and inert layer (Figure 2). The same mechanism was also applied by Whitesides et al. to develop actuators called “Hygroexpansive Electrothermal Paper Actuators” (Hamedi, 2016). The actuator inserted PEDOT:PSS into the paper to form a conducting path, which made full use of the conductivity of PEDOT:PSS and the hydrophilicity of paper to realize the hygroexpansive electrothermal actuation. To achieve a light stimuli response actuator, PEDOT, as a highly photothermal material, was transferred onto a PDMS film to compose a photothermally foldable bimorph (Lim, 2017). The active PEDOT films were used to capture near-infrared (NIR) light, which in turn generated thermal energy to control water desorption and caused the films shrinkage.

## Configurations and Fabrication Approaches

**Figure 3:**
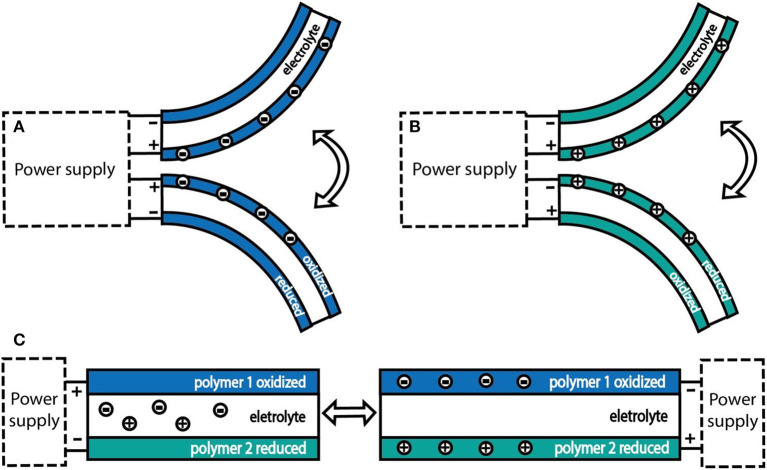
**(A)** Bending trilayer actuator made using the same “anion-driven” electrode materials; **(B)** bending trilayer actuator made using the same “cation-driven” electrode materials; **(C)** a scheme for converting bending deformation into linear deformation, using one “anion-driven” electrode material and one “cation-driven” electrode material.

Actuators with various configurations can realize different deformation, such as linear, bending, or folding (Takashima et al., 2005). The two main types of dimension changes of PEDOT-based actuators are linear and bending. Typically, self-supporting polymer films can realize slightly linear deformation, while multilayered structures can achieve macroscopic bending or linear deformation, which is caused by a relative differential expansion between layers and is more practical to applications (Figures 3A,B; Wübbenhorst and Putzeys, 2016). However, because the linear deformation is closer to the muscle deformation mode, the bending motion usually needs to be transformed into linear motion in many applications (Kaneto, 2016). One example to convert bending motion to linear motion is the use of a “three-layer” device, in which two electrode layers are made of an anion-driven film and a cation-driven film, respectively. Both electrode films take up (or expel) ions simultaneously from the electrolyte when the voltage changes, this synergistic effect leading to linear expansion or contraction (Smela, 2008; Melling et al., 2019; Figure 3C). Besides, linear motion can also be achieved by using other configurations, such as sheets, hollow fibers, etc. The methods of mass-fabricating various configuration actuators are typically spin coating, spray coating, and screen-printing techniques. In recent years, the new approaches of ink jet printing or 3D printing have developed rapidly and have been used to produce some interesting applications.

### Actuators Operating in Liquid Electrolytes

**Figure 4:**
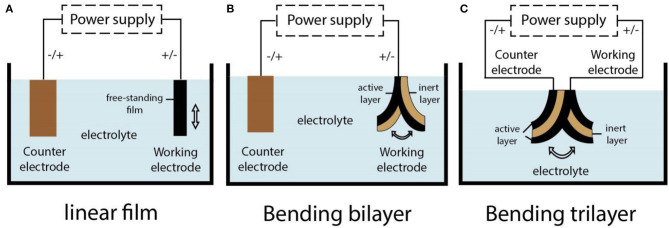
Various configurations of the PEDOT-based actuators operating in liquid electrolytes. **(A)** A model of free-standing film actuator with linear deformation; **(B)** a model of bilayer actuator with bending deformation; **(C)** a model of trilayer actuator with bending deformation.

The configurations of PEDOT-based actuators that operate in liquid electrolytes are shown in Figure 4. Linear deformation in liquid electrolytes is usually realized in the form of freestanding films (Figure 4A), of which the operating is easier, but the strain produced by a single-layer polymer film is usually small. Zainudeen et al. (Zainudeen et al., 2008) designed actuators with two or three films by electrodepositing a PEDOT(DBS) film on a PPy(DBS) film or by sandwiching a PEDOT(DBS) film between two PPy(DBS) films, which increased the stress gradient and produced greater linear strain. Bilayer configuration is typically laminated with polymer film as an active electrode layer and a porous film as an inert layer. The porous film can accommodate ions and its volume is not affected by the stimulation. During the electrochemical redox process, the volume change of the polymer film generates a stress gradient at the interface between polymer film and inert layer, which leads to the bending deformation of the actuators (Figure 4B). Gaihre et al. (2013) fabricated three bilayer actuators that were comprised of PEDOT, PProDOT, and PPy film laminated with gold-coated commercial porous PVDF film, respectively, and compared their performance in different electrolytes. The result revealed that actuators operating in organic electrolyte solutions or ionic liquids existed in completely opposite driving modes. The so-called trilayer configuration was composed of two polymer films and an inert layer sandwiched between them (Poldsalu, 2018b). One conductive polymer film was connected to the working electrode and the other to the counter electrode. When the polymer film of two electrode layers had the same ion-drive mode, one layer expanded, and the other shrunk in the reduction state. Then, the actuator produced a bending deformation. Conversely, the trilayer actuator could produce linear deformation, as mentioned earlier.

### Actuators Operating in Air

**Figure 5:**
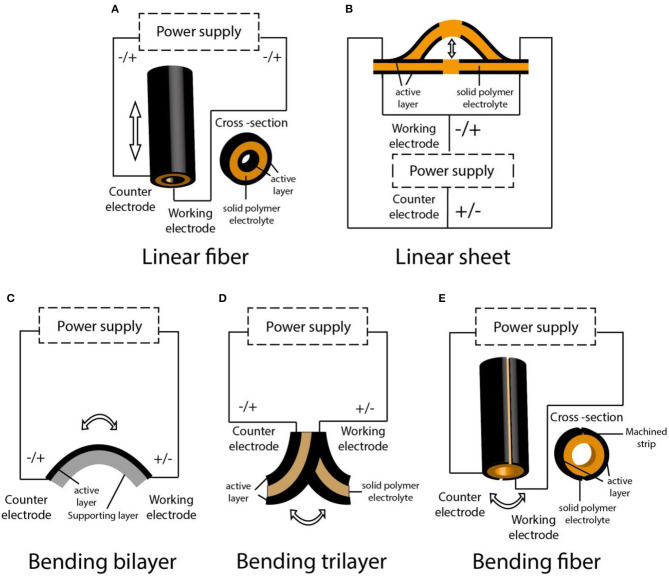
Various configurations of the PEDOT-based actuators operating in air. **(A)** A model of tubular fiber actuator with linear deformation; **(B)** a model of buckling sheet actuator with linear deformation; **(C)** a model of bilayer actuator with bending deformation; **(D)** a model of trilayer actuator with bending deformation; **(E)** a model of tubular fiber actuator with bending deformation.

The ability to operate in a humid environment is one of the advantages of actuators, especially in biomedical applications that mimic the body's fluid environment. However, as the basic component of artificial muscle, it is particularly important for actuators to be able to operate in normal atmospheric environments like natural muscle. The main configurations of PEDOT-based actuators that operated in air are shown in Figure 5. Based on the moisture sorption/desorption mechanism, various actuators with a bilayer structure can bend in air; for example, actuators that consist of multilayered PEDOT:PSS films and passive PDMS layers can realize a bending deformation in air (Taccola et al., 2015). Moreover, Greco et al. used PEDOT:PSS as a conductive layer to deposit over a liquid crystal elastomers film to fabricate a new bi-layered actuator that can bend in air through *Joule* heating (Greco et al., 2013; Figure 5C).

**Figure 6:**
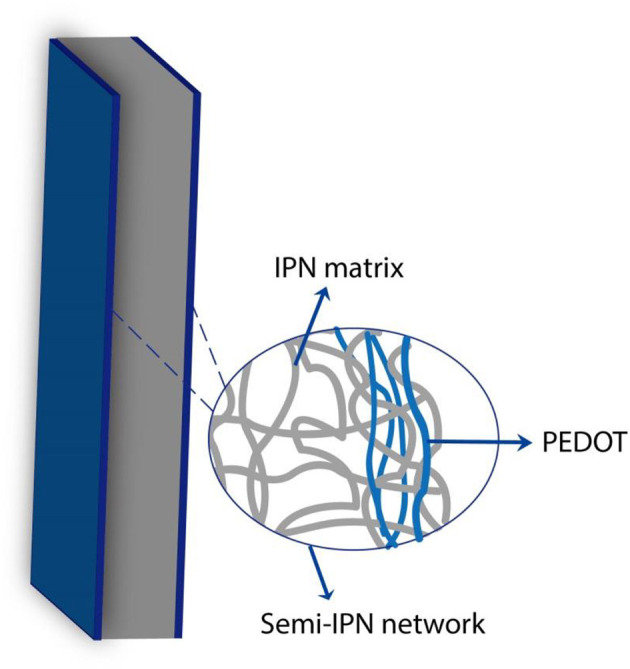
Schematic of a PEDOT-based semi-IPN system (Illustration shown the linear PEDOT embedded in an elastomeric IPN matrix).

Currently, the main configuration of PEDOT-based actuators operating in air is a trilayer structure. To cope with the problems of electrolyte volatilization and delamination, on the one hand, the solid polymer electrolyte (SPE), based on the interpenetrating polymer networks (IPNs) containing different ionic liquids as substitute for liquid electrolytes, has been used widely as the median layer (Festin et al., 2013), in which the gelatinous ionic liquid can significantly alleviate electrolyte volatilization (Armand et al., 2009). On the other hand, a semi-IPN structure based on a polymer electrode layer and SPE layer has been introduced to design trilayer actuators. This structure embeds linear conductive polymers in the elastic interpenetrating polymer network matrix to form an integrated device similar to the traditional three-layer structure (Figure 6; Vidal et al., 2002). Hollow fibers based on semi-IPNs can be driven linearly in air, as reported (Plesse et al., 2008; Figure 5A). The driving force of the device is due to the imbalance of the surface area of PEDOT electrodes inside and outside (the ratio is 1:3). The outer electrode contains a high amount of PEDOT, which acts as the counter electrode, and can easily compensate the charge from the inner working electrode in the doping/dedoping process. Therefore, compared with the inner electrode, the change in size of the counter electrode is not obvious, and the linear actuation of the hollow fiber is dominated by the deformation of the inner working electrode. Vidal and coworkers have reported another principle for building up a one-piece linear sheet actuator (Vidal, 2006; Figure 5B); through a masking technique, the polymerization of EDOT is controlled to from alternating polymerized area on the IPN substrate. The deformation of PEDOT on both sides of the un-polymerized region in the same direction during the actuation process thus causes the linear buckling of the actuator. Bending motion in air can be produced with a classical trilayer or one-piece semi-IPN structure (Figure 5D), and the output force is usually double compared with that of bilayer or freestanding film (Bar-Cohen, 2004). Madden et al. fabricated a cylindrical IPN rod-molding with a capillary tube and chemically polymerized PEDOT as an electrode layer on its surface (Farajollahi et al., 2016a,b). Then, the laser microfabrication was used to hollow out the rod to form a tube and to process the PEDOT-penetrating layer to form two machined strips on the IPN structure, which formed two independent electrodes around the cylindrical substrate. The composition of this tubular structure is similar to a typical trilayer cantilever beam structure, which can also achieve a bending motion (Figure 5E).

### Fabrication Approaches

So far, the fabrication of conductive polymer actuators has been a complex and multi-step process, involving the lamination of conductive polymer electrode film with a substrate or solid electrolyte layer and the forming technology of devices. Conductive polymers can be deposited onto substrates or SPEs by chemical and electrochemical polymerization as well as through spinning, spraying, screen printing (Lee et al., 2003), and inkjet printing techniques. Among them, inkjet printing has attracted much attention in recent years due to its non-contact deposition with substrates and advantages of not requiring a mask, additives, and complex surface treatment. This high-resolution digital printing process by CAD-drawing is convenient and efficient and is expected to achieve large-scale processing (Poldsalu, 2018a,b; Shrestha et al., 2018b). At present, actuators are formed mainly on technologies such as micro-forming (Nguyen et al., 2017), mask lithography (Taccola et al., 2013), and dry etching (Maziz et al., 2015) techniques, though most of these methods have disadvantages like requiring manual fabrication, complex post-processing, or complex assembly processes. Recently, the emergence of 3D printing has brought a new approach to the manufacture of actuators, with complex structures and high resolution that can enable rapid prototyping, customized design, and production in one step (Zolfagharian et al., 2016). However, current reports on the fabrication of electroactive polymer-based soft actuators by 3D printing technology mainly focus on ionic polymer-metal composites, dielectric elastomers, piezoelectrics, etc. There are few studies in 3D printing about conducting polymers, as conducting polymers such as PEDOT and PPy are usually not soluble in any solvent, and their soluble or colloidal dispersion, such as PEDOT:PSS and PPy-(2-ethylhexyl)sulfosuccinate (PPy-DEHS), do not attain a high electrical conductivity or mechanical properties (Maziz et al., 2016). Therefore, the doping and processing of conductive polymers and development of highly compatible dispersive conductive polymer ink may be the future direction of conductive polymer actuators for 3D printing.

## Performance Evaluation

Based on the ionic actuation mechanism, PEDOT-based actuators undergo significant net volume changes due to ion insertion or extraction during the redox process. In order to make this mass-transfer process quick, steady, and lasting, it is necessary to understand some of the basic indicators and characterizations that determine the performance of the actuators; in turn, this will guide the selection of materials and the manufacturing of the actuators (Mirvakili and Hunter, 2018). Thus, the basic and key indicators for evaluating the device performances and material properties are listed below.

### Metrics of Actuator Performances

The performance of PEDOT-based actuators can be evaluated by some basic parameters as shown in Table 1, in which the key parameters are stress, strain and speed (Smela, 2008). First, stress (σ) is usually described as the typical force that the actuator can exert per unit area, and the peak stress is the maximum force (also known as blocking force) at which a material can maintain its position in each cross-sectional area (Hara and Takashima, 2004). Blocking force is also an important parameter to describe the mechanical properties of actuators; generally, an actuator with high stiffness will produce higher blocking force (Wang et al., 2017), which is also an indispensable metric in many engineering applications. Next, strain (ε) represents the displacement in actuation direction based on the original length of the material. The value of it depends largely on the Young's modulus of materials used (Madden et al., 2004) and usually decreases with an increase in frequency (Wang et al., 2017), which is determined by the effective time of ion diffusion between electrolyte and electrodes. The stress and strain of PEDOT or PEDOT:PSS-based actuators can be characterized by a stress (σ) vs. strain (ε) curve obtained by a tensile mechanical testing machine, the value of which can be larger through doping electrodes with high mechanical materials (Kaneto et al., 2012). Last, strain rate (ε_*r*_) refers to the average strain change of the actuator in unit time during the whole driving stroke. Its value reflects the driving speed, usually up to 1–10% s^−1^, which is calculated as an average over a full bending cycle from the equation (Temmer, 2013):

$$\begin{array}{l} \varepsilon_{r}\;=\;4\varepsilon f\; \end{array}$$

where the constant 4 is the number of movements (passages) of applied potential corresponding to frequency *f* between neutral and extreme position of the whole cycle, ε is the strain difference, and *f* is the frequency of applied voltage. In addition, since the tip displacement (δ) of bending actuation is related to the strain (ε), it can be measured by a laser displacement sensor or estimated by the equation (Li et al., 2014; Nguyen et al., 2018a):

$$\begin{array}{l} \varepsilon \;=\;\frac{2d\delta }{L^{2}+\delta^{2}}\; \end{array}$$

where *d* and *L* is the thickness and free length of the actuator sample, respectively. The peak to peak displacement vs. time or frequency curves reflects the displacement variation and is usually in inverse proportion (Okuzaki et al., 2014). The peak to peak displacement vs. step voltage curve demonstrates that the degree of bending deformation is affected by the applied voltage because the ionic mobility is highly dependent on the electric potential field in the polymer matrix (Gaihre et al., 2013).

**Table 1:** Metrics of PEDOT-based actuator performances.

**Property**	**Value**	**References**
Stress (MPa)	Typically: 0–10 Max.: 50	Hara and Takashima, 2004; Wang et al., 2017
Strain (%)	Typically: 0–3	Vandesteeg et al., 2003; Vidal, 2006; Zainudeen et al., 2008; Li et al., 2014
Strain rate (%/s)	Typically: 10^0^-10^1^	Dai et al., 2010; Temmer, 2013
Life (cycle)	Typically: 10^4^-10^5^ Max.: 10^6^	Vidal et al., 2004, Kotal et al., 2016
Potential (V)	Typically: 1–3	Kim et al., 2013; Kotal et al., 2016
Conversion Efficiency (%)	Typically: <1 Max.: 1.04	Cottinet et al., 2011; Wang et al., 2017
Electromechanical coupling	Max.: 0.1	Madden et al., 2004

Moreover, the actuation durability represents the potential of practical application and can be reflected by cycle life. As reported, PEDOT-based actuators display a desirable durability that can operate to 10^6^ cycles (Vidal et al., 2004), and modifying a PEDOT electrode with high mechanical stability materials can prolong the cycle life of the actuator. Besides, the stress vs. frequency curve or strain vs. frequency curve can also reflect the cyclic stability and durability of the actuators. The scale of external stimulus needed to drive an actuator is related to the conductivity and capacitance of the material. PEDOT-based actuators can generate a motion at a lower voltage, typically about 1–3 V. Energy conversion efficiency (η) refers to the ratio of work generated to input energy, which is an important parameter to evaluate the energy utilization of the actuator, mainly depending on the electromechanical loss inside the material. PEDOT-based actuators typically have a smaller energy conversion efficiency of <1%, which is related to the elastic modulus and the output strain of actuators according to the equation (Cottinet et al., 2011):

$$\begin{array}{l} \eta \;=\;\frac{0.5Y\varepsilon 2f\times Vol}{V\times I}\; \end{array}$$

where *Y* is the Young's modulus, ε is the bending strain, *f* is the frequency of actuation, *Vol* is the volume of the actuator, and *V* × *I* is the electric input power. Electromechanical coupling refers to the proportion of input energy that is transformed into work; that is to say, the input energy in the actuator is partially converted into output work and the other part is stored in the actuator itself to generate internal mechanical energy. The electromechanical coupling thus indicates the degree of energy lost inside the actuator in addition to that which is converted to work (Madden et al., 2004). Currently, however, the electromechanical coupling of PEDOT-based actuators can only reach a maximum of 0.1, which is consistent with its energy conversion efficiency.

### Metrics of Material Properties

The performance of the actuator is often limited by the material used. Therefore, it is necessary to clarify the effect of the properties of materials as shown in Table 2 used on the performance of actuators and summarize methods to improve the properties of materials; this will help to obtain high performance PEDOT-based actuators. The conductivity of the material affects the actuator response speed directly. The high electronic conductivity of PEDOT film (10^2^-10^3^) and high ionic conductivity of ionic liquid (10^−3^-10^−2^) can accelerate the ion diffusion rate between polymer film and electrolyte, thus improving the response speed and reducing actuation voltage. The electronic conductivity (σ_*e*_) of conductive polymer electrodes can be further improved by a doping or post-treatment process. It can be measured by an in-house four-point probe, and the value can be calculated according to the Smits equation (Smits, 1958; Temmer, 2013):

$$\begin{array}{l} \sigma_{e}\;=\;I\times {\left(4.532\times w\times V\right)}^{-1}\; \end{array}$$

where *I* is the applied constant current between the outer contacts of the probe*, w* is the thickness of the polymer electrode layer, and *V* is the measured voltage between the inner contacts. Besides, Ionic conductivity (σ_*i*_) is essential to ensure good ion transport between conductive polymer electrodes and electrolytes, which can be measured by AC impedance spectroscopy with a VSP potentiostat (Biologic SA), and the value can be calculated from the real part resistance in the complex impedance diagram, according to the equation (Khaldi et al., 2012):

$$\begin{array}{l} \sigma_{i}\;=\;\frac{1}{Z}\times \frac{d}{S}\; \end{array}$$

where *Z* is the real part of the complex impedance and *d* and *S* are the thickness and the area of the sample, respectively.

**Table 2:** Metrics of material properties.

**Property**	**Value**	**References**
Electronic conductivity (S/cm)	Typically: 10–10^3^	Lee et al., 2004; Li et al., 2014
Ionic conductivity (S/cm)	Typically: 10^−4^-10^−3^	Vidal et al., 2003; Vidal, 2006; Wang et al., 2014
Capacitance (F/m^3^)/(F/g)	Typically: 10^5^-10^6^ /Typically: 10–10^3^	Kotal et al., 2016; Terasawa and Asaka, 2016; Poldsalu et al., 2017; Wang et al., 2017
Modulus (GPa)	Typically: 0–0.5 Max.: 15	Kim et al., 2013; Farajollahi et al., 2016a,b
Tensile strength (MPa)	Typically: 10–10^2^	Madden et al., 2004; Farajollahi et al., 2016a,b
Elongation at break (%)	Typically: <50	Farajollahi et al., 2016a,b; Wang et al., 2017

Cyclic voltammograms indicated the electrode reaction properties, for instance the redox characters (redox peaks or the voltage window), capacitive characters (specific capacitance), reversibility, and stability, which can be analyzed in a CV electrochemical workstation using a three-electrode system. Specific capacitance often reported use as volume specific capacitance (*C*_*v*_) and mass specific capacitance (*C*_*m*_), which refers to the charge storage per unit volume or mass at a given potential difference of the electroactive electrode. The value of it implied the capacity to uptake ions in the polymer film and can be calculated from the integrated area of CV curves using these equations (Nguyen et al., 2017; Rasouli et al., 2018):

$$\begin{array}{l} C_{v}\;=\;\int_{V_{2}}^{V_{1}}\;I\frac{dV}{\Delta V\times \vartheta \times V}\;\\ C_{m}\;=\;\int_{V_{2}}^{V_{1}}\;I\frac{dV}{\Delta V\times \vartheta \times S} \end{array}$$

where Δ*V* is the potential window, *V*_1_ and *V*_2_ are the lowest and the highest values, ϑ is the scan rate, and *V* or *S* is the volume or weight taken of the one polymer electrode of the actuators. Typically, polymer electrode materials with large specific capacitance will bring larger stress and strain to the actuator. By increasing the porosity of the material properly, the charge capacity in the polymer film will be increased and the specific capacitance can be improved (Terasawa and Asaka, 2016).

To expound the mechanical properties of the electrode films, the stress vs. strain curves can be obtained by a tensile test with a mechanical tester, which can calculate the parameters, including the Young's modulus (*E*, slope of the straight line of the stress-strain curve), tensile strength (σ_*b*_, maximum stress that the specimen can withstand before tensile fracture), and elongation at break (*e*, maximum strain rate that the specimen can withstand before tensile fracture). The electrode materials with lower Young's modulus, higher tensile strength, and elongation at break are conducive to the output of high stress and strain of actuators (Farajollahi et al., 2016a,b). In addition, conductive polymers as charge storage elements will form a double electric layer on its surface during ion diffusion, in which charge is injected and transferred. Reducing the polymer film thickness, therefore, will shorten the charge injection time. Similarly, reducing the spectator layer thickness will decrease the ion diffusion distance (the thickness of actuator typically <200 μm). Both of these in turn narrow the response time of actuators, while the output stress will be affected (Smela, 2008). Moreover, the type and ion size of the electrolyte will affect the peak strain and stress of actuators. Large ions inserted into the polymer film may cause a greater strain charge ratio, which is beneficial to produce larger peak strains. Aqueous electrolytes generally have higher ionic conductivity than organic or ionic liquid electrolytes, which helps shorten the response time. When ionic liquids are used as electrolyte materials, the high ion concentration and large ion radius in the double layer of the polymer film surface are also conducive to large peak strain and stress (Doebbelin et al., 2010).

## Actuator Materials

As previously mentioned, the properties of the electrode and electrolyte materials used greatly affect actuator performance. Doping with inorganic nanocarbon materials, small organic molecules or other high-molecule polymers can improve the electrochemical and mechanical properties of PEDOT or PEDOT:PSS electrodes, thus helping to improve the output stress and strain, durability, and energy conversion effectiveness of actuators. The construction of a solid polymer electrolyte with both ionic conductivity and mechanical stability facilitates the stable operation of the actuator in air.

### Electrode Materials

#### Pure PEDOT or PEDOT:PSS

Properly increasing the porosity in the polymer film can increase the charge density within the film, which make the electrochemical redox reaction occur quickly in the films. However, the pure PEDOT:PSS film forms too many high-porosity structures in aqueous solution, so it cannot cause deformation by ion doping under electrical stimulation (Anquetil et al., 2004). Pure PEDOT:PSS film or fiber can be subjected to local joule heating for the desorption of water in the film under the action of an electric field, resulting in linear deformation. Its strain increases with the rise of doped PSS content and relative humidity (up to 7%) (Okuzaki et al., 2009a, 2013). Besides, the PEDOT:PSS microfiber can produce more than 20 MPa stress in <0.5 s and has a cycle life up to 10^4^ cycles. This actuation mode requires high environmental humidity, and the operation is complicated (Zhou et al., 2016). It has been reported that pure PEDOT films obtained by electrochemical polymerization in an aqueous solution have linear deformation, which possesses higher stress (maximum 2.1 MPa) and strain (maximum 2%), but lower conductivity (only 8 S/cm) (Vandesteeg et al., 2003). Therefore, studies on doped pure PEDOT or PEDOT:PSS with other functional materials used them as an active electrode layer, in order to obtain bilayer or trilayer actuators with better performance.

#### Composite With Nanocarbon Materials

Pure PEDOT or PEDOT:PSS films can be doped with additives to improve electrical conductivity, capacitance, and mechanical properties. Nano-carbon materials, including carbon nanotubes, graphene, and activated carbon aerogels, have good electrical conductivity, mechanical rigidity, and chemical stability, which can be used as ideal additives for electrode materials. The single-walled carbon nanotube (SWCNT)-doped PEDOT:PSS electrode shows a synergistic effect of electrostatic double-layer capacitance and faraday capacitance, so that the composite electrode has a larger specific capacitance than the pure PEDOT:PSS electrode (from 22 to 244 F/g). Meanwhile, single-walled carbon nanotubes form highly entangled mesoporous networks in the composite polymer film, which increases the Young's modulus of the electrode film (from 102 to 214 MPa), and corresponding actuators are also proven to produce output stress three times higher than the undoped ones (Terasawa and Asaka, 2016). Carboxylic multi-walled carbon nanotubes (MWCNTs) have also been reported to improve the conductivity and mechanical properties of PEDOT:PSS electrodes. The PEDOT:PSS electrode with 30% carboxylic MWCNTs has an electrical conductivity that is one order of magnitude larger than the original electrode (from 4.64 to 153.75 S/cm) and output strain increases to 0.64%. The tensile modulus increases by eight times that of its original value (from 127.64 to 918.27 MPa), which makes the energy conversion efficiency as high as 1.04% (Wang et al., 2017). This is because the highly conductive MWCNTs network in the polymer matrix increases the electrical conductivity and mechanical strength, and the π-π interaction between MWCNTs and the PEDOT chain as well as the hydrogen bonding between MWCNTs and PSS chain causes the charge to be transferred from the PEDOT chain to the MWCNTs, which is beneficial to ion migration and promotes the conversion efficiency of electrical energy to mechanical energy. Heteroatom sulfur and nitrogen-doped reduced graphene oxide have a larger specific surface area, better ion transport performance, and mechanical properties. The composite electrode obtained after doping with PEDOT:PSS has a higher capacitance and conductivity (up to 505 F/g, 767 S/cm), and the mechanical flexibility and stability increase under bending deformation. The constructed trilayer actuator has a maximum bending strain of 4.5 times higher than that of an undoped one at the voltage of 1 V, and the durability is improved (remain 96% strain after 18,000 cycles; Kotal et al., 2016). The addition of activated carbon aerogel particles in PEDOT:PSS is beneficial to ion transmission and large elastic modulus of the electrode, which increases the specific capacitance and output stress while interfering with the PEDOT:PSS structure, resulting in a decrease in strain caused by the pure Faraday current in the polymer film. Therefore, the mixed electrode is suitable for the actuator requiring high stress (Poldsalu et al., 2017).

#### Composite With Other Polymers

PEDOT or PEDOT:PSS films composite with other polymers can significantly improve their mechanical property. For instance, the electrochemical polymerization of PPy(DBS) film and the electrodeposition of PEDOT(DBS) on its surface to form the actuator with double-layer composite film exhibits electrochemical characteristics similar to that of pure PPy film in electrolyte, and its strain in a high scan rate between reduced state and oxidation state is improved effectively (up to 3%) without any delamination phenomenon (Zainudeen et al., 2008). Moreover, the double-network hydrogels based on PEDOT:PSS hydrolyzed partially to realize bending motion in an aqueous electrolyte with a pH > 4.7 by electrical stimulation (the strain up to 66% in 120 s). In the double-network hydrogels, poly(acrylamide) (PAAm), as the first network converts the amide group into a carboxyl group through hydrolysis and ionization, makes the matrix negatively charged and can respond to electrical stimulation. The semi-IPN hydrogel based on PEDOT:PSS serves as the second network, in which the conductive PEDOT phase and PSS polyelectrolyte phase improve the electronic and ionic conductivity of the hydrogels, respectively. Besides, the PEDOT phase forms clusters when compression fractures, which can disperse local stress and increase the mechanical strength of double-network hydrogels (Dai et al., 2010).

#### Modification by Organic Small Molecules

The conductivity of PEDOT:PSS electrode film can also be improved by modifying it using an organic solvent. Doping of PEDOT:PSS film with polar solvent DMSO (volume ratio: 4:1) can promote the separation of the PEDOT chain and PSS chain in the polymer matrix so that the conductivity of the PEDOT:PSS film at room temperature increases to 43 S/cm. The doped electrode laminated with an active PVDF layer can be used to prepare a bimorph actuator that can run stably under a high frequency electric field (Lee et al., 2004). It has been reported that electrode films with high conductivity and stretchability can be obtained by doping (50%wt) xylitol with PEDOT:PSS films and subsequently exposing it to heat treatment. First, the doped xylitol can form hydrogen bonds with PSS chains to weaken the hydrogen bonds between the PSS chains, thus reducing the viscosity between colloidal particles. Next, it can function similar to a plasticizer to enhance the flexibility and stretchability of the film. Meanwhile, it also has the additional ability to increase carrier mobility between colloidal particles and improve the conductivity of thin films (up to 407 S/cm), and the constructed trilayer actuator can generate 0.15% strain under the stimulation of 2 V voltage (Li et al., 2014).

### Solid Polymer Electrolyte Materials

The development of high-performance electrode materials can be used in a variety of configurations, including self-supporting polymer films, bilayers, trilayers etc. As for trilayer actuators, to achieve stable operation in air requires an appropriate electrolyte layer. In addition to the development of high-performance electrode materials, therefore, the development and modification of electrolyte materials and the optimization of ion migration in the separation layer is also important for the development of high-performance actuators. Traditional solid electrolyte materials used commercial poly (vinylidene fluoride) (PVDF) porous film soaked with ionic liquid. Its porosity ensures the ionic conductivity and its relatively large Young's modulus (~70 MPa) ensures mechanical stability, though its size tailoring is restricted (Schmidt et al., 2006; Gaihre et al., 2013). Synthesized composite solid-polymer electrolyte materials with adjustable ion conductivity and mechanical properties are therefore of great significance in practical applications of actuators.

#### IPN Based on Polyethylene Oxide

Ionic conductive polymer polyethylene oxide (PEO) is often used to form an interpenetrating polymer network, with mechanically strong polymers such as polycarbonate (PC), polybutadiene (PB), nitrile butadiene (NBR), and polytetrahydrofuran (PTHF) as a separation layers, and soak with ionic liquid to form solid polymer electrolyte materials (Cho M. et al., 2007; Cho M. S. et al., 2007; Plesse et al., 2007). The PEO is an ionic conductive polymer in which the oxygen atom can form coordination bonds with the metal cation in the electrolyte to promote ion pair separation, facilitate ion transmission, and improve ion conductivity. However, it also has significant disadvantages, such as a high degree of crystallization at room temperature and the fact that ion transport mainly occurs in the amorphous region of the polymer; reducing the crystallinity of PEO is therefore conducive to ion migration. Vidal et al. introduced short poly (ether oxide) side chains into the PEO network, which significantly reduced the crystallinity of PEO, thus increasing the volume available for ion migration, and improved the ionic conductivity of the branched PEO network to 5 × 10^−4^ S/m (Vidal et al., 2003). Since both PEO and PC networks contain ether oxygen units, the two networks have good compatibility, and are thus conducive to the formation of interpenetrating networks. On the other hand, due to the interaction between the ethylene oxide unit of PEO and dioxyethylene unit of EDOT, the PEO network has an affinity to the monomer EDOT. The composite PEO/PC film was soaked in EDOT solution and the polymerization of PEDOT within it to obtain an integrated semi-IPN actuator with a conductivity of 10 S/m (Vidal et al., 2003). Moreover, the PEO formed interpenetrating networks with NBR or PTHF, and their ionic conductivity could reach 10^−3^ S/m after soaking in the ionic solution (Plesse et al., 2011). The actuator with PEO/NBR/PEDOT configuration could achieve 2.4% strain at 2 V voltage (Vidal, 2006).

#### Natural Macromolecule Cellulose

As an electroactive material, cellulose has advantages of light weight, biodegradation, and operation under low voltage. Using cellulose electroactive material containing a lithium chloride solution as an intermediate layer electrolyte, PEDOT:PSS forms a nanometer coating on both sides, which can improve the durability of the trilayer actuator and significantly reduce (90%) energy loss (Mahadeva and Kim, 2009). Bacterial cellulose is produced by all sorts of bacteria and has higher chemical purity and crystallinity, tensile strength and stiffness, and an improved ultra-fine network structure compared with plant cellulose. The freeze-dried bacterial cellulose forms a porous structure with high strength and high Young's modulus (15 GPa); it can absorb a large amount of ionic liquid and has enhanced electrochemical and mechanical properties, and it is thus suitable for use as an ion exchange film. The trilayer actuator composed of PEDOT:PSS electrodes and the freeze-dried bacterial cellulose can produce a displacement of 1.5 mm at 1 V voltage (Kim et al., 2013). In another example, polyvinyl alcohol (PVA) is also a water-soluble, biodegradable, and biocompatible polymer. Gelatinous bacterial cellulose cannot be dispersed in water but can be converted and decomposed into individual nanofibers by the oxidation of TEMPO. By crosslinking PVA with oxidized bacterial cellulose, hydrogen bonds were formed through interaction with hydroxyl groups in PVA and cellulose, and thus the composite film is obtained. Compared with pure oxidized bacterial cellulose film, the crystallinity and stiffness of composite film is reduced, and the ionic conductivity of composite film is increased by 20%, which increased the maximum bending displacement of the actuator by 2.3 times its original value (Wang et al., 2014).

## Applications

### Bionics and Biomedicine Applications

So far, PEDOT-based actuators have been widely used in bionics or biomedicine, intelligent textiles, microactuators, and functional applications. Actuators that work within the field of bionics or biomedicine are interesting and meaningful. A semi-IPN trilayer actuator with a PEDOT and PEO/NBR host matrix was integrated into a biomimetic vision system by Vidal and coworkers (Festin et al., 2011). In this system, the position of the micro-camera is controlled by a closed loop formed by a trilayer actuator excited by a pulse-width modulation signal and a visual perception board. The function of actuators on both sides of the micro-imager—to adjust the imaging position—was similar to that of the ciliary body in the eye. The same group also reported a new application of PEO/NBR/PEDOT semi-IPN films, which was integrated into a biomimetic perception system as an artificial tentacle to imitate the tactile perception of a rat tentacle (Festin et al., 2014). The artificial tentacle consisted of a PMMA fiber and two semi-IPN films where one acted as an actuator to derive the tentacle movement and another as a sensor to sense and collect real time deformation during the actuation process. What's more, continuous progress in the development of hybrid muscle was generated by combining skeletal muscle and artificial structures. Kim et al. Kim et al. (2016) developed a hybrid muscle powered by C_2_C_12_ skeletal muscle cells based on the PEDOT/MWCNT hydrophilic sheet to achieve biomimetic actuation, which was controlled by electric field stimulation and presented flexible movement and physical durability in solution. The PEDOT/MWCNT sheet has excellent biocompatibility, mechanical stability, and shape adjustability, which make it ideal as a platform for culturing a variety of cells to form mixed muscles in the field of biomedicine and is expected to be used as a patch for artificial organs or biosensors.

### Textiles and Wearable Applications

**Figure 7:**
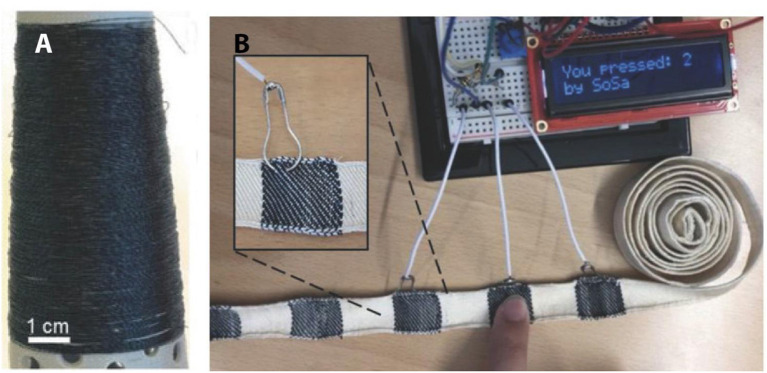
**(A)** Photograph of a yarn bobbin with 100+ m of dyed yarn; **(B)** photograph of the textile capacitive touch sensor with key number 2 activated. The insert shows the safety pin electrical connection. **(A,B)** Reproduced with permission (Terasawa and Asaka, 2016). Copyright 2018, Wiley.

When used in textiles and other wearable applications, the actuator generates mechanical motion, sound, or substance release (such as thermal energy) upon receipt of electronic signals. With the development of smart electronic textiles, great advances have been made in the actuator for wearable applications such as textile-capacitive touch sensors, wearable heaters and artificial muscles etc. For the electronic textiles, an essential component is electrically conducting yarns. Wet spinning PEDOT:PSS fiber has played an important role in the development of electronic textiles due to its inherent electrical conductivity and charge-storage capability. The wet spinning process, with a novel and one-step route, eliminated the need for post-spinning treatment with organic solvent such as ethylene glycol, as was reported by Wallace and co-workers. From this, fibers can be imparted enhanced electrical conductivity and redox cycling properties (Jalili et al., 2011). Lund et al. also developed conducting silks; they used PEDOT:PSS as ink to coated over mulberry silk yarns and post-treated them in a dimethyl sulfoxide (DMSO) or ethylene glycol (EG) solvent, which improved their conductivity to the highest level for coated fiber: 70 S cm^−1^(Figure 7A). They also produced a woven band that could serve as a capacitive touch-sensitive textile keyboard using dyed PEDOT:PSS-conducting yarns, which connected with a liquid crystal display that displayed messages when pressing the PEDOT:PSS textile area (Lund et al., 2018; Figure 7B). Similarly, the coating of fabrics with a PEDOT-PPy layer was synthesized by a chemical electrochemistry method to form textile actuators, and the strain can be improved 53-fold that of pure fabrics. This makes it possible to integrate them into textiles and garments to assist walking, and there is great potential for development in driving intelligent prostheses and the applications of soft robots (Maziz et al., 2017). Moreover, as we know, electrical impulses in the human heart can be detected by placing electrodes at specific points around the human heart. To this end, Kimura et al. presented a foldable textile based on PEDOT:PSS and poly(vinyl alcohol) (PVA)-blended fiber as a flexible electrode that has enhanced conductivity and mechanical strength to detect human heartbeats (Miura et al., 2014). This will provide a platform to realize integrated, wearable electronics.

### Microapplications

In recent years, the rapid development in the production of actuators that operate in air or solutions has been demonstrated on a micro scale. Various types of microactuators have been applied in many fields such as mechanics, chemistry, biomedicine, etc. (Jager et al., 2000). Among several smart electroactive materials, conducting polymers have attracted much attention because of their ability for miniaturization, which improves the electrochemical and mechanical properties of microactuators, such as speed and stress output. Taccola et al. fabricated a bilayer microactuator that operated in solution with the structure of PEDOT:PSS coated with SU8 photoresist. This was patterned by lithography technology to form microfingers that can bend in the electrochemical redox process (Taccola et al., 2013). A PEDOT-based microactuator that can work in air was reported in the early years. Composed of SPE with an IPN structure and PEDOT electrodes interpenetrated on both sides, the plasma dry etching technique was used successfully on the extremely thin (12 μm) conductive microactuator film (Khaldi et al., 2011). In order to overcome the shortcomings of manual operation in some steps of microactuator production, Maziz and coworkers reported a top-down integration process to micro-fabricate soft, conducting polymer actuators to achieve continuity in direct synthesis, patterning, and operation (Maziz et al., 2016). Through this innovative and sequential layer stacking, the trilayer microstructures were fabricated by using two PEDOT electrodes by vapor-phase polymerization and the PEO/NBR semi-interpenetrating polymer network as the intermediate SPE layer. These materials have been successfully patterned using photolithography and reactive ion etching techniques and are easily scalable for the simultaneous batch-production of microactuators with hundreds of electromechanical structures (Nguyen et al., 2018b).

### Other Functional Applications

**Figure 8:**
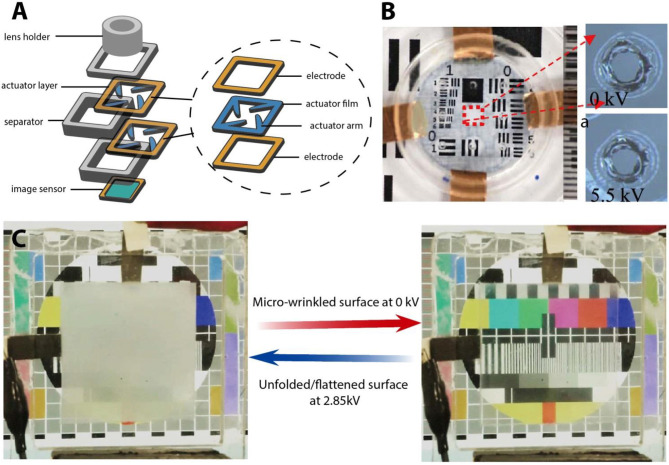
**(A)** Micro autofocus lens actuator that uses bending PEDOT-based conducting polymer actuators; **(B)** a photograph of a two-layer MPDEA and micrographs showing the hole contraction upon activation. Reproduced with permission (Anquetil et al., 2004). Copyright 2018, ACS. **(C)** Smart window based on electric unfolding of microwrinkled TiO_2_ nanometric films, switching between hiding or revealing a Philips pattern on a liquid crystal display. Reproduced with permission (Okuzaki et al., 2013). Copyright 2018, ACS.

Various functional applications have been realized by many PEDOT-based actuators with different configurations. Lee et al. fabricated a flexible organic film speaker using PEDOT:PSS (DMSO) as an electrode and PVDF as an active layer by using the screen printing method, which showed a flat and stable sound pressure level over 400 Hz (Lee et al., 2003). Ikushima et al. prepared a micro autofocus lens actuator by using trilayer-bending PEDOT:PSS (PEO)/PVDF/ PEDOT:PSS (PEO) actuators and simply drip-coating PEDOT:PSS (PEO) solution onto the treated PVDF membrane; they designed the tests to inspect the actuator's use in micro autofocus lenses, showing that the actuators can operate stably for more than a million cycles (Ikushima et al., 2010; Figure 8A). PEDOT or PEDOT:PSS can be combined with other electroactive polymers such as a dielectric elastomer to design actuator with new features to accommodate new applications. Shrestha et al. developed a transparent and adjustable sound-absorption material based on a micro-perforated dielectric elastomer actuator with an inkjet-printed PEDOT:PSS electrode, which can adjust the absorption spectrum while transmitting light. Such a transparent absorber is thus suitable for noise absorption on window glass (Shrestha et al., 2018b; Figure 8B). Moreover, a smart window based on small-strain microwrinkling/unfolding to switch between frosted and clear was created by sandwiching a dielectric elastomer actuator between a pair of thin, microwrinkled TiO_2_/ PEDOT:PSS film electrodes (Shrestha et al., 2018a). This all-solid-state smart window was durable and high-performance at a low cost and low power, which exceeded even that which was reported by a commercial polymer-dispersed liquid crystal smart window, as shown in Figure 8C.

## Conclusion and Outlook

The high conductivity of PEDOT and the soluble processability and flexibility of its dopant, PEDOT:PSS, means actuators based on them are able to provide a large driving strain at low voltage while also being lightweight, of high durability, and of low cost. The PEDOT-based conductive polymer actuators attracted the attention of researchers back in 1996 and have since made great progress in actuator applications owing to its admirable intrinsic properties. This feature article has provided an overview of the basic actuation mechanisms, performance evaluation criteria, configurations, processing technologies, recent development of materials, and applications of PEDOT-based actuators. However, although many studies have shifted the actuators from principle verification to practical applications, there are still many scientific and technical challenges to overcome before widespread commercialization can occur.

Further developments on both the device materials and applications of soft actuators as artificial muscles may revolve around the wearable themes. To be incorporated into wearable and flexible electronics, the selection of material should have enough strength, structural rigidity, and flexibility, and it should be able to provide a sufficient amount of actuating force; in particular, it should possess comfortability and biocompatibility. Conducting hydrogels based on PEDOT:PSS may become potential applications in future development. The trilayer PEDOT-based actuators are the main configurations that produce significant deformation, but the fact that they work in the air causes certain problems, one of which is that the ionic liquid will leave the device over time and will be substituted by the environment molecules. Hence, it is important to add an appropriate encapsulation layer to protect the electrolyte's solvent from evaporation or the ionic medium from being lost when the device is in operation (Rinne et al., 2019; Takalloo et al., 2019). Moreover, actuator applications in the future should be energy saving and environmentally friendly; a new study presented of an autonomously powered actuator that can bend reversibly in both directions and is driven by glucose and O_2_ only (Mashayekhi Mazar et al., 2019) provided a perfect demonstration of future applications. Finally, developing compatible inks to use in 3D printing technology for the mass production of actuators will provide a powerful impetus to their commercial applications.

## Author Contributions

This study was conceived and designed by JX and BL. The paper was drafted, written by FH and revised, approved by BL and YX.

### Conflict of Interest

The authors declare that the research was conducted in the absence of any commercial or financial relationships that could be construed as a potential conflict of interest.
